# Evaluation of melanin production by *Sporothrix luriei*

**DOI:** 10.1590/0074-02760170339

**Published:** 2018-01

**Authors:** Ingrid Ludmilla Rodrigues Cruz, Maria Helena Galdino Figueiredo-Carvalho, Rosely Maria Zancopé-Oliveira, Rodrigo Almeida-Paes

**Affiliations:** Fundação Oswaldo Cruz-Fiocruz, Instituto Nacional de Infectologia Evandro Chagas, Laboratório de Micologia, Rio de Janeiro, RJ, Brasil

**Keywords:** Sporothrix luriei, L-tyrosine, pyomelanin

## Abstract

There is a paucity of studies on the cell biology of *Sporothrix luriei*, the less common of the pathogenic *Sporothrix* species worldwide. The production of DHN-melanin, eumelanin, and pyomelanin were evaluated on the mycelial and yeast forms of the *S. luriei* ATCC 18616 strain. The mycelial form of this species produced only pyomelanin, which protected the fungus against environmental stressors such as ultraviolet light, heat, and cold. The yeast form was unable to produce any of the tested melanin types. The lack of melanin in the parasitic form of *S. luriei* may be an explanation for its low frequency in human infections.

From 1898 to 2006, sporotrichosis was attributed to a single species *Sporothrix schenckii* (Kauffman 2006), or its variety, *S. schenckii* var. *luriei* (Padhye et al. 1992). With the advance of polyphasic fungal taxonomy, the single so-called *S. schenckii* species was separated into four species: *S. schenckii* sensu stricto, *Sporothrix brasiliensis*, *Sporothrix globosa*, and *Sporothrix mexicana* (Marimon et al. 2007). Moreover, *S. schenckii* var. *luriei* was elevated to the species level, and it is now called *Sporothrix luriei* (Marimon et al. 2008).

The first documented *S. luriei* infection occurred in 1956 (Ajello & Kaplan 1969). Three other human sporotrichosis cases related to *S. luriei* have been reported (Mercadal-Peyrí et al. 1965, Alberici et al. 1989, Padhye et al. 1992). The main diagnostic feature in these cases was the presence of fungal eyeglasses-like cells (Padhye et al. 1992). A case in a dog, diagnosed through molecular methods, has also been reported (Oliveira et al. 2011).

Different from other *Sporothrix* species, the absence of sessile dark-pigmented conidia has been described for *S. luriei* (Marimon et al. 2008). *Sporothrix* pigmentation is the consequence of melanin deposition in the fungal cell wall (Almeida-Paes et al. 2017). Melanins are present in the three major pathogenic species of the genus: *S. brasiliensis*, *S. schenckii*, and *S. globosa* (Almeida-Paes et al. 2015), and they protect these species against several stress conditions that they can face in the environment and during parasitism. Moreover, genomic data have revealed that melanin biosynthesis in *S. schenckii* and *S. brasiliensis* is similar (Almeida-Paes et al. 2017). To the best of our knowledge, there is no information about melanin in the *S. mexicana* cell wall. Since it was reported that *S. mexicana* produces dematiaceous conidia, as does *S. schenckii* and *S. brasiliensis* (Marimon et al. 2007), the black pigment observed in *S. mexicana* conidia is also thought to be related to melanin deposited in the cell wall of this species.

The lack of melanin in *S. luriei* is a possible hypothesis for its low prevalence in human infections. Therefore, this study aimed to determine whether this species can produce the three major types of fungal melanins (DHN-melanin, eumelanin, and pyomelanin) under well-established *in vitro* conditions used to study melanisation in other *Sporothrix* species.

The *S. luriei* strain INCQS 40253 (ATCC 18616 / CBS 937.72) was used in this study. The *S. brasiliensis* type strain (CBS 120339) was included as a control for melanin production. Strains were maintained in the mycelial form in Sabouraud dextrose agar at 25°C and in the yeast form in brain heart infusion agar at 35°C. Production of DHN-melanin was assessed in a minimal medium (29.4 mM KH_2_PO_4_, 10 mM MgSO_4_, 13 mM glycine, 15 mM dextrose, 3 µM thiamine, pH 5.5). Experiments to detect eumelanin and pyomelanin were performed in minimal medium supplemented with 1 mM L-dopa or 10 mM L-tyrosine, respectively. Tricyclazole (16 mg/L), glyphosate (100 mM), and sulcotrione (16 mg/L) were used to supplement the media to evaluate the blockage of the DHN-melanin, eumelanin, and pyomelanin metabolic pathways, respectively (Almeida-Paes et al. 2009, 2012, Teixeira et al. 2010).

Both the mycelial and yeast forms of *S. luriei* and the control *S. brasiliensis* strains were tested for melanin production at an initial inoculum concentration of 1 × 10^6^ conidia or yeasts/mL in the above described media. Fungi were incubated in the dark for 15 days at 25°C (conidia) or 35°C (yeasts) on a rotary incubator at 150 rpm. To detect DHN-melanin or eumelanin, cells were harvested from the cultures described above and washed three times in phosphate-buffered saline (PBS) and suspended in 1 M sorbitol/0.1 M sodium citrate solution. Protoplasts were generated by incubating cells at 30°C in a solution containing 10 mg/mL cell wall-lysing enzymes (from *Trichoderma harzianum*; Sigma Chemical Co.) for 1 h at room temperature. Protoplasts were washed with PBS and incubated in 4.0 M guanidine thiocyanate for 1 h at room temperature with frequent vortexing. The resulting material was washed three times in PBS and boiled in 6.0 M hydrochloric acid for 1 h. Supernatants of cultures supplemented with L-tyrosine were filtered through 0.22-μM membranes, acidified to pH 2.0 using 0.5 M hydrochloric acid, and left for 24 h at room temperature. The precipitated pyomelanin was harvested by centrifugation (12,800 × *g*) and resuspended in sterile distilled water.

As expected, the control *S. brasiliensis* strain produced the three melanin types in both morphologies, as described previously (Supplementary data, Figure). In contrast, the chemical treatment with enzymes, denaturant, and hot acid dissolved *S. luriei* mycelial and yeast cells without generating dark particles retaining the shape and size of the conidia, hyphae, or yeast cells (Fig. 1A). However, small dystrophic particles, similar to those observed when the DHN-melanin synthesis was blocked by tricyclazole in *S. brasiliensis* or *S. schenckii*, were observed in both fungal morphologies, even in the absence of this inhibitor (Fig. 1B). The *S. luriei* yeast form was also unable to produce pyomelanin under the *in vitro* conditions employed herein. However, supernatants of *S. luriei* mycelial cultures supplemented with L-tyrosine turned black after 10 days of growth at 25°C (Fig. 1C). This pigment was acid resistant, and its synthesis was specifically blocked by sulcotrione, thereby confirming this pigment to be pyomelanin.

**Fig. 1:**
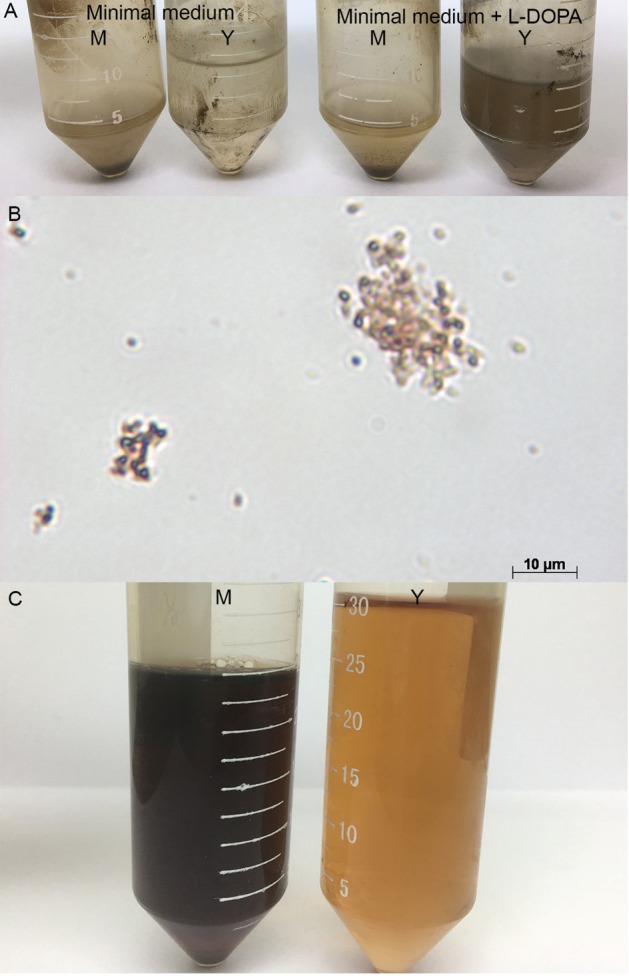
evaluation of melanin production by *Sporothrix luriei*. (A): macroscopic aspects of hot-acid resistant particles yielded by mycelial (M) and yeast (Y) cells grown in minimal medium and minimal medium supplemented with 1 mM L-DOPA; (B): microscopic aspects of the hot-acid resistant particles, bar: 10 µm; (C): black-soluble pigment observed in the supernatant of the mycelial (M) culture in minimal medium supplemented with 10 mM L-tyrosine and absence of the pigment in the culture supernatant of the yeast culture in the same medium.

Since the *S. luriei* mycelial form produced pyomelanin, we hypothesised that this pigment would be involved in protection against harsh environmental conditions. To check this hypothesis, *S. luriei* conidia were harvested from cultures with and without L-tyrosine, adjusted to 1 × 10^8^ conidia/mL, and submitted to either 15, 30, 45, 60, or 75 seconds of ultraviolet (UV) light (290 µW/cm^2^). In addition, conidia were incubated for 24 h at 38°C and stored without cryoprotectants at 4°C for six months to evaluate heat and cold protection, respectively. Six measurements were taken in each of these experiments. The results were analysed with the Mann-Whitney test using GraphPad 5 software. As depicted in Fig. 2A, melanised conidia submitted to UV light had more colony forming units than non-melanised conidia (p < 0.05). Moreover, only melanised conidia survived UV exposures longer than 60 s. Melanised *S. luriei* conidia were also more resistant to heat and cold (p < 0.05 for both experiments) than non-melanised cells (Fig. 2B).

**Fig. 2:**
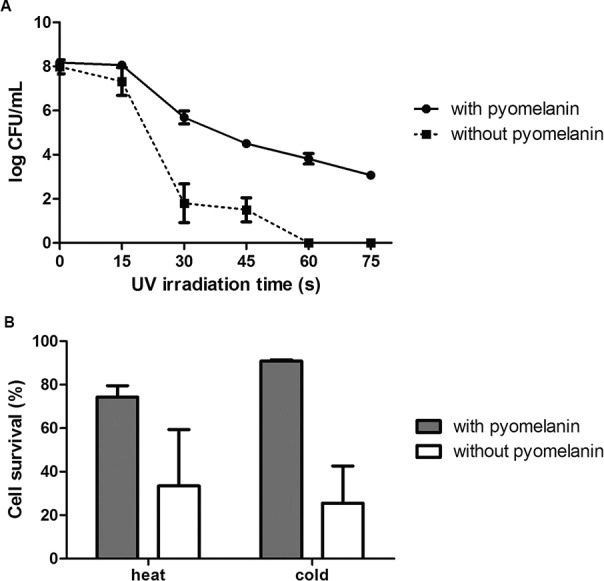
resistance of melanised *Sporothrix luriei* conidia against environmental stressors. (A) growth (CFU/mL; mean ± standard deviation) of cultures with and without pyomelanin after ultraviolet light irradiation for different exposure times; (B) percent survival (mean ± standard deviation) of conidia with and without pyomelanin after heat (38°C) and cold (4°C) exposures. For all conditions, p < 0.05.

The presence of pyomelanin in the mycelial form of *S. luriei* may be a result of the better adaptation of this species to environmental conditions, which agrees with the protection that this pigment confers to the fungus against abiotic stress factors. The degree of protection against UV radiation observed in this study was similar to that observed with *S. brasiliensis* pyomelanin and other fungal melanin types (Almeida-Paes et al. 2012).

Melanins were not found in the *S. luriei* yeast cell wall. Its low incidence as an agent of sporotrichosis (Zhang et al. 2015) and the requirement of a high *S. luriei* inoculum to achieve virulence in an experimental infection model using the same strain as in the present study (Fernández-Silva et al. 2012) may result from the lack of melanin in the parasitic form of this species. Under the same conditions that other *Sporothrix* species are able to produce DHN- and eumelanin (Almeida-Paes et al. 2009), only small acid-resistant particles that did not have the shape and size of *S. luriei* cells were observed. Besides the three melanin types studied in this work, some fungi produce other pigments, such as γ-glutaminyl-3,4-dihydroxy-benzene-melanin, catechol melanin, *p*-aminophenol melanin, deoxybostrycoidinmelanin, and asp-melanin. The observed particles are not likely to be related to these uncommon types of fungal melanins, since they are expressed in sexual reproduction structures and/or require exogenous compounds for production (Toledo et al. 2017). The black acid-resistant structures of *S. luriei* are similar to those produced by *S. schenckii* and *S. brasiliensis* when the DHN-pathway is inhibited with tricyclazole (Almeida-Paes et al. 2009), suggesting that melanin synthesis in *S. luriei* is blocked by an unknown mechanism. These dysmorphic particles resemble the melanosome-like structures observed in *S. schenckii* (Almeida-Paes et al. 2017). One hypothesis is that they are polymerisation products of accumulated intermediary metabolites of a hindered melanin synthesis pathway. Since information on the whole genome of *S. luriei* is unavailable, a search for mutations or missing genes related to melanin synthesis was not possible.

Due to the paucity of available *S. luriei* strains (Marimon et al. 2008), we were able to study melanisation in only one strain. This was also a limitation in other important studies on *S. luriei* taxonomy and virulence (Marimon et al. 2008, Oliveira et al. 2011, Fernández-Silva et al. 2012). Future studies with more strains are necessary to gain a better understanding of *S. luriei* cell biology and pathogenesis.
