# Supplementary figures and images for: Evaluation of melanin production by Sporothrix luriei
Source: Mem Inst Oswaldo Cruz. 2018 Jan;113(1):68–70. doi: 10.1590/0074-02760170339 (PMC5719544; doi:10.1590/0074-02760170339)

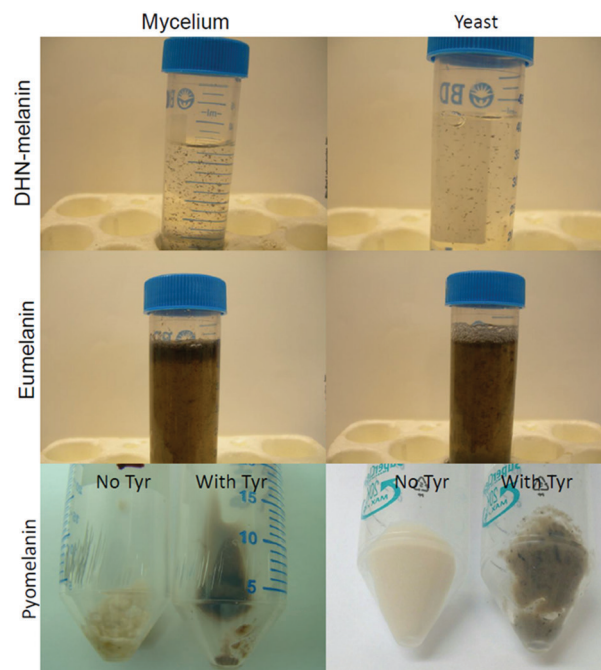

Production of three types of melanin by *Sporothrix brasiliensis* CBS120339.

Supplement: Supplementary file 1 [file 0074-0276-mioc-113-01-0068-Suppl01.pdf]
